# IR Imaging of Solid Lubricant Coatings on Concealed/Disjointed Surfaces for Transparent Polymer Delivery Device Applications

**DOI:** 10.3390/s20226408

**Published:** 2020-11-10

**Authors:** Anton Walsh, Natalia Rebrova, Steven Darby, Killian Barton, Raymond Wolfe, Finbarr Buckley, Liam Lewis, Michael McAuliffe

**Affiliations:** Centre for Advanced Photonics and Process Analysis, Cork Institute of Technology, T12 P928 Cork, Ireland; Natalia.Rebrova@cit.ie (N.R.); Steven.Darby@cit.ie (S.D.); Killian.Barton@cit.ie (K.B.); raymond.wlf@gmail.com (R.W.); Finbarr.Buckley@cit.ie (F.B.); Liam.Lewis@cit.ie (L.L.)

**Keywords:** IR transmission imaging, multispectral imaging, thin film coating evaluation, polymer delivery devices, solid lubricant coating, polypropylene, poly(vinylpyrrolidone), polyurethane, process monitoring

## Abstract

Transparent polymer delivery devices often contain a solid lubricant coating on a stronger bulk polymer. The distribution of lubricant coating must be monitored for device optimisation appraisals and to ensure consistency during mass production. However, coating evaluation is difficult to perform as surfaces are often concealed and/or disjointed. Dye stain analysis, which is destructive and time-consuming, is the current industry standard. We present a prototype IR transmission microscope to evaluate micron-level coating coverage of polyurethane and/or polyvinylpyrrolidone on a poly(propylene)-based delivery device. The device has a common industrial configuration, containing a duct and bevel. Inferred absorption of the coating was used to identify coating coverage and a multivariate analysis was used to remove the effects of absorption and scattering by the bulk. Coverage on concealed and disjointed surfaces was imaged and evaluated from a single camera viewpoint and ≈50 μm defects were detectable. The industrial applicability of the prototype was demonstrated using comparisons with dye stain analysis by estimating water dilution of coating and identifying artifacts in coating, which may indicate machine malfunction. The sensitivity and speed of the IR technique makes it a favourable alternative to the current industry standard.

## 1. Introduction

Transparent polymer-based delivery devices often contain a solid lubricant coating of a substance such as polyethylene glycol (PEG), poly(hydroxyethyl methacrylate) (PHM), poly(styrene-b-isobutylene-b-styrene) (PSIT), or poly(vinylpyrrolidone) (PVP), on a stronger bulk polymer material, such as such as poly(ethylene) (PE), poly(carbonate) (PC), or poly(propylene) (PP) [1,2]. The coating may also have antimicrobial, water-repellent, or adhesive functions [1,2]. The distribution of lubricant, by spraying, dipping, brushing, and/or chemical/physical vapor deposition [3,4], must be monitored during device optimisation appraisals and to ensure consistency during mass production.

PP, the bulk material of the delivery device presented, is the second most widely produced commodity polymer plastic. PP’s main application is in packaging of consumer products; however, applications can be found in most commercial fields [5]. In delivery devices, its mechanical stability, transparency, bio-compatibility, functionality, durability, and safety make it a popular component [6]. The PP bulk material is coated with PU- and PVP-based films for lubrication. PVP’s diverse properties, including chemical stability, non-toxicity, adhesive, electrical, solubility in water and organic solvents, and high capacity to form interpolymer complexes, make it a highly versatile product. The capability to form complexes with many active substances has made it a common component in tablets, granules, pellets, soft gelatine capsules, gels, hydrogels, films, and coatings. In the case of coatings, its hydrophobic properties make it a popular lubricant [7]. PU forms the cross-linker between the mechanically stable PP and PVP lubricant. Commercially, foam is the most common form of PU manufactured and can be easily tailored to be rigid or flexible. As a binder, PU offers good adhesion, low chemical reactivity, efficient drying, low temperature flexibility, and adequate scratch tolerance [8].

An abundance of thin film coating evaluation techniques for presence, thickness, and density are available [9]. Scanning electron microscopy (SEM) or transmission electron microscopy (TEM) can have sub-micrometer resolution and clear results, using such add-ons as energy-dispersive X-ray spectroscopy (EDX), but a cross-section of the sample is required and thickness can only be measured in one dimension [10]. Profilometry, either stylus or optical, contains high spatial resolution, but recording a thickness requires a full-depth step in the coating [11]. Gravimetric measurements can measure the total quantity of coating, but they do not contain any coverage detail. Acoustic wave techniques can be employed, but they are limited due to the similar mechanical properties of the plastics involved. Similarly, magnetic techniques cannot be used on plastics.

Optical techniques are popular, as they offer non-destructive characterisation. Raman and fluorescence microscopy [12,13] use laser excitation and spectroscopic molecular fingerprints to identify samples. Surface imaging can be made by placing the sample on a three-axis translation stage and an image is formed in a stepwise manner, with spatial resolution at the micron level. However, depth analysis to measure coating thickness is difficult and depends on many factors, such as the opaqueness and Raman/fluorescence quenching properties of the different materials. Even with satisfactory optical properties of the sample, the stepwise approach makes the measurement time-consuming and not suitable for high-quantity industry allotments. Similarly, FTIR microscopes use a three-axis translation stage and have even lower temporal resolution due to the translation of interferometer mirrors/beamsplitters [14]. Other spectroscopic techniques include optical coherence tomography (OCT) and reflective techniques, attenuated total reflection (ATR) and ellipsometry [9,15]. A limitation of spectroscopic techniques is the requirement of direct access/sight of the coating. In reflective techniques, thin films concealed beneath the bulk are not observable without destroying the sample and disjointed surfaces require time-consuming sample re-alignment. In the case of a delivery interface, such as a duct with a bevel tip, both concealed and disjointed surfaces are present.

Dye stain test methods can evaluate coating coverage over a large area and in combination with microscopy can have high spatial resolution [16,17,18]. The technique is destructive, so lot quality assurance sampling must be used during manufacturing. Concealed surfaces can be studied, if the bulk sample is transparent in the visible. The technique has several limitations; it is destructive, time-consuming, and reveals little detail, and some dyes are carcinogenic. However, the simple visible identification of the presence of coating on a non-planar sample make it a useful tool, especially where samples are easily disposed. Dye stain testing is regularly applied to devices containing transparent thin film coatings, such as PEG, PHM, PSIT, PVP, and polyurethane (PU), and can be considered the industrial standard. Although transparent in the visible, these thin films have molecular absorption fingerprints in the IR, where commonly used bulk materials, such as PE, PC, PP, and polystyrene (PS) are transparent. Consequently, a transmission microscope in the IR has the advantages of dye stain testing without the destructive, time-consuming, and hazardous dye deterrents. Measurement of the absorption of thin film coating also provides more qualitative and/or quantitative information.

We describe an IR transmission set-up which interrogates coating coverage on a nozzle with a common configuration for a delivery device head, an elliptical duct with a bevel tip. The nozzle contains coating on both concealed and disjointed surfaces, demonstrating the technique can be easily expanded to other delivery device shapes. The PU/PVP coatings employed and PP bulk material of the sample are commonly used in industry. Their IR absorption properties are shared with other polymer coatings, such as PEG, PHM, and PSIT, and bulk materials, such as PE, PC, and PS, and so the technique can easily be expanded to include other common materials.

The sample delivery device interface is described in full in Section 2.1. The transmission microscope, consisting of a SiC globar, filter wheel, imaging optics, and microbolometer array detector, is described in Section 2.2. IR absorption/scattering fingerprints are used to identify bulk and coating transmission independently. The process of separating bulk and coating transmission properties on the duct and bevel of the sample is given in Section 2.3. Measurements of coating coverage on samples are compared with dye stain and SEM measurements in Section 3. In Section 4, the applicability of the set-up to industrial samples is discussed. Tests for coating coverage, water dilution, and machine malfunction identification are made using the set-up.

The set-up combines multispectral imaging, IR absorption spectroscopy, and machine vision to measure sub-micron level coating coverage of PVP and PU on the surface of a 200 μm thick PP wall. Coating coverage on concealed and disjointed surfaces are identified on a single image, without the need for re-alignment of the sample, with a spatial resolution of ≈50 μm. A conclusion and outlook for the technique is presented in Section 5.

## 2. Materials and Methods

### 2.1. Polymer Delivery Device

**Figure 1:**
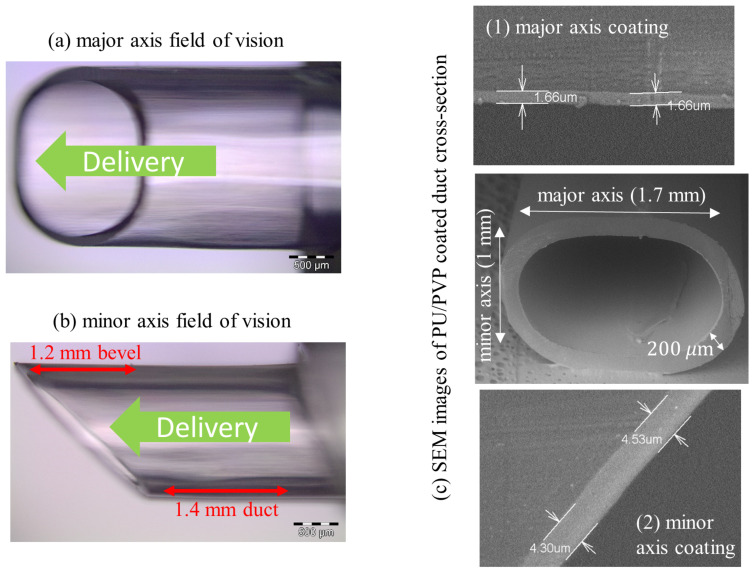
Sample delivery device interface: elliptical duct with a bevel tip. The inner wall of the tubing is coated with a thin film coating of a combination of PVP and PU. The PP body and PVP/PU coating are transparent in the visible. Darkfield reflection images from a visible microscope with a ×5 objective of (**a**) major and (**b**) minor axes views are shown on the left. (**c**) SEM images of a cross-section from the duct is shown on the right. The thickness of coating at selected segments of the (1) major and (2) minor axes of an industrially coated sample is also shown. Coating thickness is highest on the minor axis.

Images of the sample delivery device nozzle interface are shown in Figure 1 (All samples were supplied by Alcon Laboratories Ireland Ltd., Cork, Ireland). The main body of the nozzle is an injection-molded PP polymer. The nozzle is split into two regions for analysis: duct and bevel. An SEM cross-section of the duct is also show in Figure 1. The duct has an elliptical shape, with major and minor access sections of ≃1.7 and ≃1 mm. Only analysis of the major axis is presented here. The set-up can be adapted to include measurements of the minor axis by introducing the option of mounting the sample at 90 to the present set-up alignment. For details of minor axis measurements and comparison with the major axis, see [19]. The duct length is ≃1.4 mm, with the bevel extending out along one side of the major axis. The bevel has a curved rim, with a maximum extension of ≃1.2 mm located at the centre of the major axis. The PP wall thickness is 200 μm. The nozzle inner walls are coated with a PVP-based thin film, a common industrial lubricant to improve delivery. Between the PP body and PVP coating, a PU-based thin film is employed as an adhesion promoting layer. The combined thickness, *t*, of the PVP/PU coating on the PP substrate is 1≤t≤5μm, for an appropriately coated nozzle. Thickness is a function of position on the nozzle. In the duct segment, *t* is higher on the minor axis.

### 2.2. IR Transmission Microscope Design

The sample is illuminated with a stabilised 24 W silicon carbide globar source (dimensions: 209×55×57.5 mm), Thorlabs SLS203L. The source power peaks at ≈4350 cm^−1^ and, after collimation, has an approximate peak power of 100 μW/nm over a circular area of diameter D≃2.2 cm. The nozzle is placed on a custom-made mount, with the major axis of the nozzle duct perpendicular to the IR probe beam. An achromatic lens, f=10 cm, images the globar onto the nozzle. The globar is a suitable extended source, as the ≈0.3 aspect ratio of the source surface imaged on the nozzle is similar to the ≈0.4 aspect ratio of the nozzle. IR transmitted through the nozzle is focused onto a 480×640 micro-bolometer array (PhoenixCore Thermal Imaging Camera, Visimid Technologies) using an achromatic lens, f=7.5 cm. A filter-wheel is placed before the camera for wavelength selection. Two wavenumber regions can be selected at ≈3735 cm^−1^ and ≈3385 cm^−1^ (see Section 2.3.1). The camera records unsigned 16 bit (U16) greyscale images of IR intensity. Ambient temperature signal and source IR intensity for both wavenumber ranges are measured prior to each measurement. All measurements consist of the average of 100 frame captures acquired at 10 Hz.

The optics are custom chosen to offer a depth of field (DOF) just over the length of the minor axis of the nozzle, enabling imaging of surfaces on opposing sides of the nozzle simultaneously on the single camera array. Each pixel covers a ≈11 μm square of surface, equivalent to the resolving power of the optics, R≈13μm.

**Figure 2:**
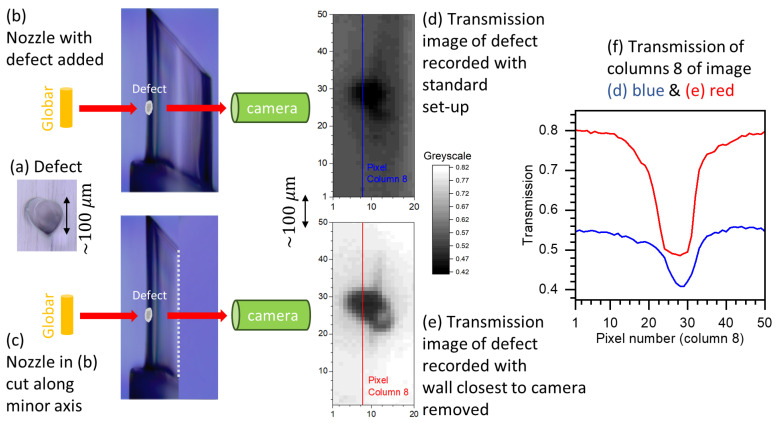
Determining reduction in image quality on surface farthest from camera due to opposite wall; see text for detailed description. Defects as low as ≈50 μm in size are identifiable.

Image quality of the inner wall coating is reduced as the probing IR is also transmitted through two 200 μm PP walls. To determine the reduction in image quality and the minimum size defect identifiable on the inner wall surface farthest from the camera, a ≈100μm pinprick was placed on the surface and imaged under different conditions. A visible light microscope image of the defect is shown in Figure 2a, along with set-up schematics (Figure 2b,c) and transmission images (Figure 2d,e). A transmission image at ≈3735 cm^−1^ of the defected nozzle was measured under normal conditions, see schematic Figure 2b and image Figure 2d. Without removing the sample from the mount, the nozzle was split, leaving only the wall containing the defect. A transmission image was measured, see schematic Figure 2c and image Figure 2e. The contrast in the second image (Figure 2e) is higher, as removing a wall increases the baseline intensity of IR across all pixels. The extent of blurring can be accessed by comparing comparable columns of pixels from both transmission images. The transmission of a column of pixels along the center of the defect is shown for both images in Figure 2f. The extent of blurring appears negligible and coating defects of the order of ≈50 μm are identifiable on both walls. Both surfaces are imaged simultaneously on the same pixel array and, consequently, the wall where a defect occurs cannot be identified. However, for most industrial applications, identifying the presence of defects is satisfactory.

### 2.3. Imaging of Coating Transmission

#### 2.3.1. Methodology for Isolating Coating Features from Bulk

**Figure 3:**
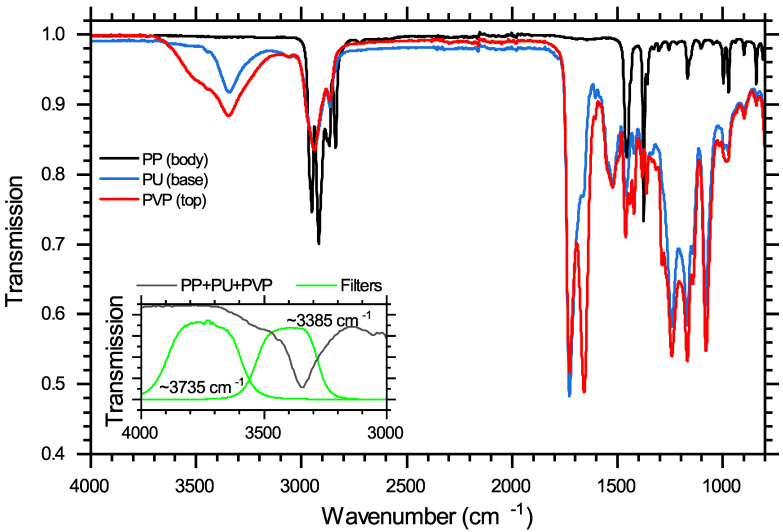
ATR transmission spectra of separate PP, PU, and PVP material samples. The insert shows transmission spectra of a full coated nozzle bevel tip and filters employed for measuring transmission in the range of PU/PVP absorption, centred at ≈3385 cm^−1^, and in the range where only scattering by PP/PU/PVP occurs, centred at ≈3735 cm^−1^ [19].

ATR transmission spectra of separate PP, PU, and PVP material samples are shown in Figure 3. The penetration depth of the evanescent wave is ≃2 μm. PP has no absorption features in two regions where features of PVP/PU occur, a broad amine feature in the shortwave IR (SWIR), at ≈3385 cm^−1^, and a carbonyl stretch feature further into the mid-IR (MIR), at ≈1700 cm^−1^ [20,21]. The MIR absorption is significantly stronger, ×7.5 at peak, compared to the SWIR feature. The micro-bolometer detector has similar absorbance in both wavenumber regions. However, the luminosity of the globar is six times stronger in the SWIR region. The combination of the weaker luminosity of the globar and the stronger absorption made it impossible to measure coating thickness levels of t>3μm using the MIR range, whereas the absorption at ≈3385 cm^−1^ was found to be strong enough to identify sub-micron level coating thickness and contained enough SWIR signal intensity to measure the larger coating depths. IR light is narrowed to the absorption feature range using a bandpass filter placed before the camera; see the insert of the Figure 3 for the transmission spectrum. The transmission spectrum of a PU/PVP-coated nozzle in this range is also shown, demonstrating the overlap in filter transmission and the absorption fingerprints of coatings.

Although the PP nozzle bulk does not absorb at ≈3385 cm^−1^, optical losses will occur due to scattering. To estimate these losses, transmission is measured at ≈3735 cm^−1^, where both the PVP/PU coatings and PP bulk do not absorb. See the insert of Figure 3 for selected filter transmission spectrum. The filter centre is shifted only ≈350 cm^−1^ from the absorption feature centred filter and possesses a similar bandwidth and transmission maximum. On average, the PP wall thickness is more than fifty times greater than the coating thickness. A linear calibration function of optical loss of the bulk at ≈3385 cm^−1^ as a function of optical loss at ≈3735 cm^−1^ is determined by measuring transmission images of uncoated nozzles. Using this linear relationship, we can predict optical loss of PP at ≈3385 cm^−1^ on a coated nozzle by measuring transmission at ≈3735 cm^−1^ and can subsequently remove this quantity from the total loss at ≈3385 cm^−1^, isolating the PVP/PU absorption losses.

#### 2.3.2. Calibration of Optical Losses Due to PP Nozzle Body

**Figure 4:**
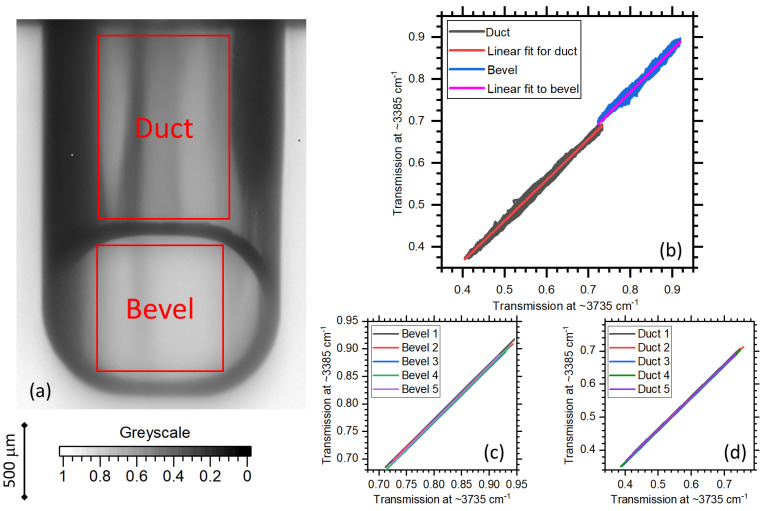
(**a**) Image of transmission at ≈3735 cm^−1^ of an uncoated nozzle. (**b**) Measured transmission at ≈3385 cm^−1^ as a function of ≈3735 cm^−1^ for duct and bevel components of uncoated nozzle. The regions where data is extracted is highlighted in (**a**). Linear fits to data from both components is also shown in (**b**). Linear fits to five sample uncoated nozzles is shown for the bevel in (**c**) and duct in (**d**).

The nozzle duct and bevel components are calibrated separately. An U16 greyscale image of transmission, where 0% transmission is 0 bits and 100% transmission is 65,535 bits, of an uncoated nozzle is shown in Figure 4a. Plots of measured transmission at ≈3385 cm^−1^ as a function of transmission at ≈3735 cm^−1^ and linear fits for duct and bevel components of an uncoated nozzle sample is shown in Figure 4b. The selected regions where data was extracted is marked in Figure 4a. A calibration cannot be found for the whole surface of the duct or bevel, as the scattering relationship becomes non-linear approaching the edges of the major axis due to the changing angle of incidence. The width of duct and bevel measured is 1.2 mm along the major axis. Linear fits to five other sample uncoated nozzles are shown in Figure 4c,d for bevel and duct, respectively. Transmission at ≈3385 cm^−1^ can be estimated to within ±2.5% using transmission at ≈3735 cm^−1^.

Transmission at ≈3735 cm^−1^ is also used to determine any defects in the bulk PP material. This can be used to separate where defects are created, during the coating procedure or from pre-coating nozzle damage.

## 3. Results

A machine vision Python 2.7 algorithm, employing the OpenCV package [22], is used to identify nozzle boundaries in ≈3385 cm^−1^ and ≈3735 cm^−1^ transmission images, such as Figure 4a, in order to extract duct and bevel components. The OpenCV *canny edge detection* function is applied to the U16 greyscale ≈3735 cm^−1^ transmission images to find the outlines of the nozzle. The subsequent application of *findContours* function provides an array of contours which form the outline. The algorithm calls the *drawContours* function to draw the contours on the U16 greyscale image with a 3-pixel-wide line. Subsequently, the algorithm finds coordinates for the borders by doing a horizontal and vertical scan of the image. From border coordinates, it calculates the centers of the nozzle’s bevel and duct. The size of the areas of interest are determined during calibration and the algorithm forms a rectangular crop based on the calculated centers and the width and height provided as input parameters. The parameters determined using ≈3735 cm^−1^ transmission image are subsequently used to crop the ≈3385 cm^−1^ transmission image. The full version of the code can be found in the Supplementary Material, along with sample ≈3735 cm^−1^ transmission images, Figure S1, of before and after cropping, and of contour outlines. A custom 3D printed mounting device was crucial to ensure the nozzle edges were perpendicular and alignment within <50 μm was reproducible.

**Figure 5:**
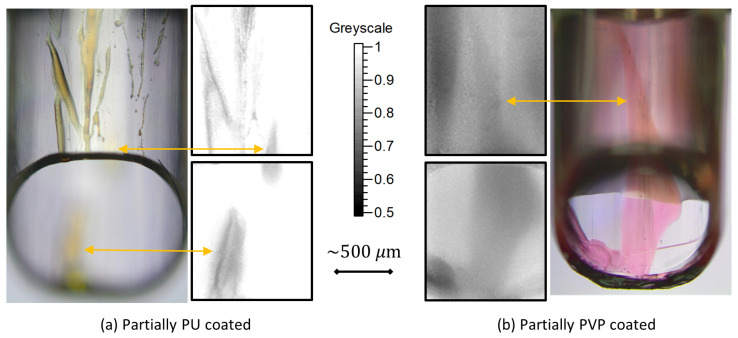
Nozzles partially coated with PU (**a**) and PVP (**b**). For (**b**), the nozzle was coated with PU beforehand using the standard industrial treatment. The U16 greyscale images represent transmission of the coating measured using the IR transmission microscope. Absorption and scattering effects of the bulk PP are not present in the greyscale images. The dye stain images were recorded using a ×5 objective.

Figure 5a shows images from a nozzle partially coated with PU. The greyscale image shows coating transmission measured using the IR transmission microscope. After measurement, a Cresol dye solution was used to test for the presence of PU [23] and a visible image of the dyed nozzle is also shown. Patterns in coating identified using the dye stain are matched in the transmission greyscale images. The dye stain images were recorded using a ×5 magnification and a DOF less than the width of the nozzle. Consequently, the sample position must be adjusted to identify patterns on opposing wall surfaces. For the IR image, in contrast, patterns on both inner walls of the major axis of the duct and on the surface of the bevel can be measured with the single sample position.

Figure 5b contains images from a nozzle partially coated with PVP. The nozzle was coated with PU beforehand using a standard industrial process. Similar to Figure 5a, an IR transmission image of coating was measured first, followed by a dye stain analysis. A Congo red solution was employed to identify the presence of PVP [24]. The presence of coating is seen throughout the IR greyscale images, due to the presence of the PU base coating. The pattern in PVP coating can be identified by gradients in the reduction of transmission, which is relatively constant for purely PU coated industrial samples. These patterns are matching with patterns observed in the dye stain image.

**Figure 6:**
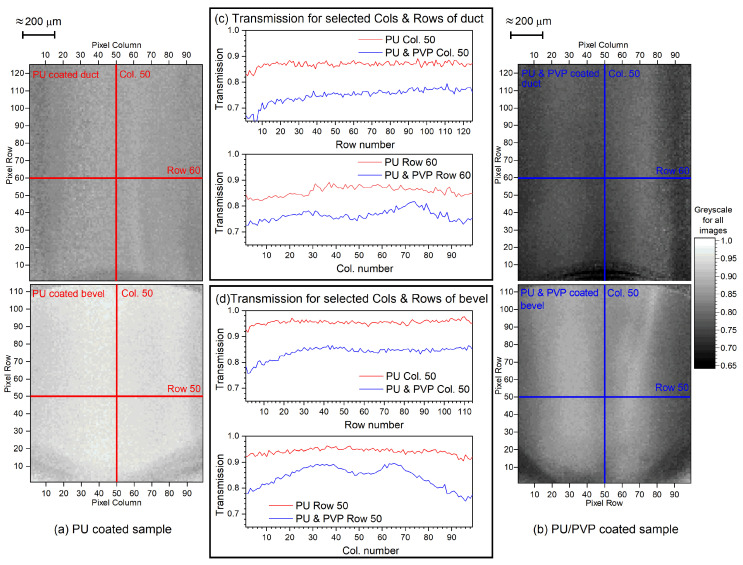
IR transmission greyscale images of PU-coated (**a**) and PU/PVP-coated (**b**) nozzles. Changes in transmission along selected columns and rows, highlighted in the greyscale images of both nozzles, are shown for the duct (**c**) and bevel (**d**).

Figure 6a shows PU samples and Figure 6b shows PU/PVP samples where coating was applied using standard industrial processes. Coating coverage has several treatment parameters, including pre-plasma treatment, PU and PVP quantities, dispersion mechanism and curing. Comparisons between coating properties measured using IR transmission and other techniques, SEM and dye stain, are the focus of the present work and no discussion of coating treatment performance will be made. Figure 6c,d contains plots of coating transmission as a function of pixel number along selected columns and rows, in approximately the center of the axes. For purely PU-coated samples, the transmission for selected columns/rows are relatively consistent, varying by only 3–4%. The only exception being approaching the rims of the bevel and duct, where decreases in transmission occur, see col. 50 coating transmission plots for duct and bevel. There were no resolvable differences in coating thickness on SEM measurements of solely PU-coated samples. Thickness of <1 μm was found [19].

Reduction in transmission from PU- to PU/PVP-coated samples was observed as expected. Compared to solely PU-coated nozzles, general features in PU/PVP coating were seen. A gradual decrease in transmission along the delivery axis towards the bevel was observed in the duct and, similar to the PU-coated samples, a further increase in coating thickness was seen towards the rim; see col. 50 coating transmission plot for duct. An increase in coating thickness along the delivery axis was also seen from SEM imaging. Slight patterns were seen perpendicular to the delivery axis on the duct, as well as transmission decreases towards both sides, and, at the centre, a shallow hollow in the coating exists; see row 60 coating transmission plot for duct. The increase in thickness towards the edges was confirmed by SEM images, where coating along the centre of the major axis is typically <2 μm and on the centre of the minor axis is >4 μm. The shallow hollow in coating was not observed from SEM imaging or in dye stain testing. This is not surprising for dye stain testing, as only the presence of coating can be measured. For SEM imaging, the nozzle must be sliced. Cutting the nozzle along the delivery axis is relatively smooth and the risk of damage to the coating is slight. Cutting perpendicular to the axis is more difficult and damage to the coating is more likely. Consequently, the slight hollows observed in the IR images would be hard to reproduce using SEM.

The bevel showed more pronounced patterns than the duct for PU/PVP-coated samples. The patterns were similar as a function of position to the duct, with increased coating towards the rim of the bevel along the delivery axis, as well as increased coating towards both edges perpendicular to the delivery axis. The increase in coating thickness along the delivery axis matched SEM imaging, where coating up to ≈5 μm can be measured at the outer rim. The hollow at the centre of the major axis is more pronounced on the bevel.

SEM imaging of the surface morphology of industry standard coated samples identified area roughness at sizes below ≈50 μm, our determined resolution, down to features encompassing a few micron. Estimating the associated profile roughness of coated samples with SEM imaging is difficult, as the sample must be cut to observe a cross-section. Random cross-sections of coated nozzles revealed continual fluctuations of coating thickness of up to ≈0.2 μm on cross-section lengths of ≈50–60 μm; see Figure S2 of the Supplementary Material. Fluctuations in coverage on this scale are not identifiable with the present configuration of the IR transmission microscope, as the smallest possible defect size detectable is ≈50 μm. The smoothing of local defects, below the microscope resolution, is used to estimate a ≈0.01 noise level in transmission. Quantum statistics could not be used to determine noise in transmission, as the microbolometer camera does not measure light intensity directly, but a change in temperature. Instead, noise in transmission was estimated using areas where a low change in transmission, ≤0.2, is viewed over surface cross sections of >200 μm, and fitting a low-order polynomial. The noise was then estimated from the residuals of the fit.

While other techniques, such as SEM imaging, optical profilometery, and Raman/fluorescence microscopy, can offer higher resolution coverage measurements, their limitations on quantifying coverage depth, added to their time constraints, make them inapplicable for bulk sample appraisals in an industrial setting. The industry standard dye stain technique, which the presented technique targeted for replacement, employs visible microscopes to manually scrutinize dyed samples. Using a standard laboratory microscope, a technician can identify defects down to a ≈5 μm size. The higher resolution is compromised by the narrow DOF, compared to the presented IR transmission microscope, which increases the time required to scrutinise samples. We demonstrate for the ≈2 mm sized delivery device presented that the lower ≈50 μm resolution is not needed to create a test equivalent to the dye stain test, which has superior resolution (see Section 4.1). In some applications, minimum allowed size defects may not be established. More crucial is the relative thickness of the coating, above or below the optimum, which cannot be easily identified using dye staining testing and can be easily identified with the IR transmission microscope.

## 4. Discussion

The merit of the IR transmission technique in industrial settings is presented for three applications. In Section 4.1, a test to identify deficient coating using IR images was compared to the standard dye stain test analysis. In Section 4.2, quantitative measurements of optical density were used to predict dilution of coating solution by water. In Section 4.3, the use of IR images for qualitative determination of machine malfunction was discussed.

### 4.1. Comparison to Dye Stain Testing

Dye stain testing can be considered the standard validation test for coating coverage of PU and PVP. Although subjective, time-consuming, and destructive, the ability to identify defects in the visible make it a popular tool. Random lot assurance sampling is used to ensure quality during manufacturing. An automated test to replace dye stain testing for PVP was developed using the IR transmission microscope. The test was created from IR coating transmission images of 96 PU/PVP-coated nozzles sampled from different industrial production runs which have passed the dye stain test procedure. Dye stain testing was performed using a Congo red solution [24] and samples were examined by eye under a microscope, using up to ×7 magnification, for the absence of dye colour. Nozzles were inspected from several fields of view, including the minor axis walls which are not viewed using the IR microscope presented.

The IR transmission image test was based on the accumulative results of individual pixels. For the 96 recorded images, coverage at corresponding pixels was assumed to approximate a normal distribution. Average transmission, μ, and standard deviation, σ, in transmission for corresponding pixels were calculated. The requirement for individual pixels was set as: coating transmission, *T*, must be T<3σ+μ. If the pixel overlaps with an appropriately coated region, then there is a >99.7% probability the pixel will pass the requirement. For the overall nozzle to pass the IR test, no more than 115 pixels can fail the individual test. The value of 115 pixels was determined empirically to make a tight test and remove any possible error which may occur from divergence of transmission from a normal distribution at a pixel.

A motorized translation stage Thorlabs LTS300, with a 47 μm position reproducibility, was added to the IR microscope set-up and a custom mount capable of holding eight nozzles was 3D printed. Results for eight nozzles could be acquired in ≈3 min, significantly improved from the greater than ≈20 min required for dye stain analysis.

The efficiency of the test was evaluated by sampling 72 nozzles, 64 from production runs with 100% coating efficiency and 8 from coating runs with inferior process settings and a 100% fail rate. The set-up identified all nozzles from the failed run and failed one nozzle which passed the dye stain test.

**Figure 7:**
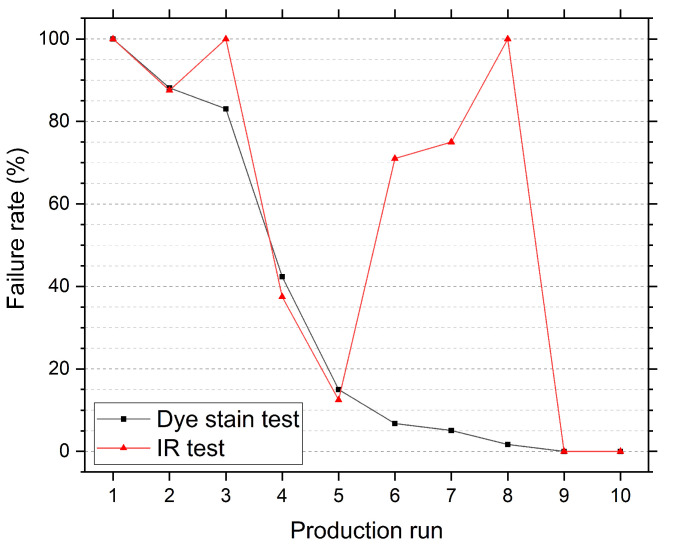
Failure rates measured using IR transmission and dye stain testing for 10 consignments of samples produced using different process settings, such as rotation mechanisms and coating solutions.

A comparison more closely analogous to industrially situations, where a sudden 100% fail rate is unlikely in a run and gradual changes in machine functionality lead to a stepped increase in fail rate, was made. Production runs using different process settings, including rotation mechanisms and coating solution, were made to create consignments of nozzles with differing fail rates, determined using dye stain analysis. Ten production runs were made. One consignment contained a 100% fail rate, six contained partial fail rates between 5% and 90% and two had a 0% fail rate. Figure 7 shows a comparison between the fail rates from dye stain and IR transmission testing. Eight samples from each run were tested using the transmission microscope. The transmission microscope successfully identified all the consignments containing fails. Consignments which contained a 0% and 100% fail rates from dye stain testing, the two extremes, had corresponding results using the IR technique. Although the IR transmission microscope test identified all consignments which contained fails, the fail rate can differ considerably to dye stain results. A correlation could not be found between a setting of the coating process and divergence in fail rates. As dye stain testing is subjective, matching fail rates is unlikely. In general, the IR transmission test gave a higher fail rate than the corresponding dye stain test. By the absence of subjectivity, the IR test can identify system degradation faster than dye stain analysis. The present set-up is easily up-scaled to facilitate the testing of every nozzle in production and not just a subset. However, the requirements of the pass threshold may need to be adjusted when considering mass production.

### 4.2. Application to Water Dilution

**Figure 8:**
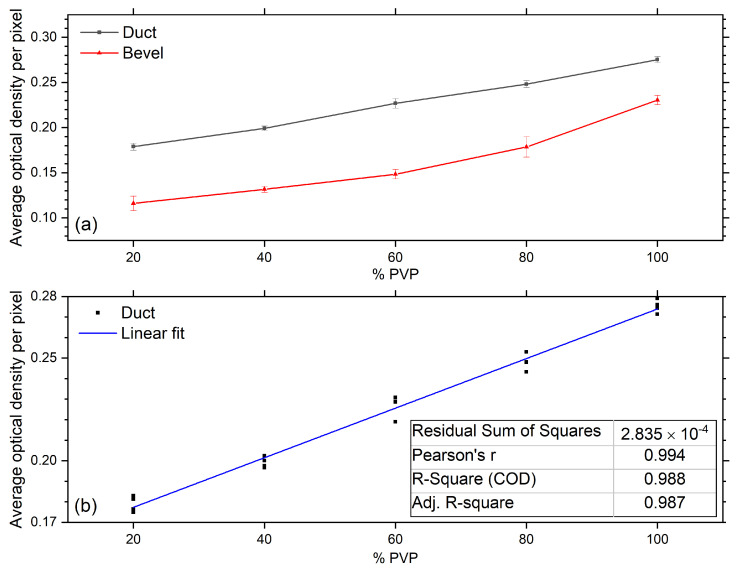
(**a**) Average optical density per pixel, for duct and bevel components, of samples coated with diluted PVP solutions. (**b**) Linear fit to average optical density per pixel data of duct. The fit parameters are given in the inserted table and demonstrate water dilution could be monitored using IR transmission imaging.

In an industrial setting, dilution of the coating solution may occur inadvertently during cleaning processes such as line flushing. To simulate undermining of the coating solution, four diluted concentrations, 20%, 40%, 60%, and 80%, of PVP by water were made and used as coating solution. The nozzles were PU-base-coated as normal. Four samples at each concentration were made up. The average optical density (OD) per pixel for each nozzle on the duct and bevel was used as an indicator for water dilution, see Figure 8.

The error in OD for each concentration is estimated as the standard deviation. The average OD of pixels in the duct is lower than the bevel, as OD of the duct is from two PVP layers. However, the average OD of a single layer on the duct is weaker than the bevel. This is due to the increasing coating thickness along the delivery axis as you move to the exit, seen with IR transmission images in Figure 6 and SEM measurements. The OD on the bevel has more variation compared to the duct and can be seen from the standard deviation. The average OD on the duct shows a linear trend with concentration, R-square = 0.988 for fit (see Figure 8b). Consequently, the dilution of coating solution could be monitored effectively using IR coating transmission images of the duct.

### 4.3. Identification of Machine Malfunction Using Machine Vision

**Figure 9:**
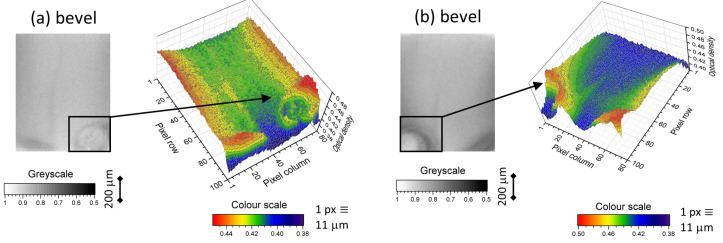
Two examples, (**a**,**b**), of bubbles of air forming, between coating and bulk, on bevels. The bubble artefacts can be observed in 2D greyscale images by ring structures or as hollowed-out circular swells in OD in 3D images.

A statistical approach was used to identify deficient nozzle coating and water dilution was identified from linear trends in an average OD per pixel. Machine vision can be used to identify patterns which are associated with a specific type of machine malfunction and, consequently, can efficiently locate where the source of the problem is occurring. An example of this approach is the identification of bubbles, an undesirable artefact of air trapped between coating and the bulk wall. These can be separated from other coating features, using the stronger absorption visible on the side walls of the bubble. In 2D transmission images, this appears as a ring or in 3D images of OD; as a hollowed out circular swell, see Figure 9.

## 5. Conclusions and Outlook

We measured the coverage of common thin film coating products, PVP and PU, on 200 μm thick walls of a PP-based delivery device. The device has a common configuration, composing of an elliptical duct and bevel. The transparency of PP at ≈3385 cm^−1^ enables the study of PU/PVP coverage on concealed surfaces inside the tube, and a long DOF enables the imaging of opposing inner surfaces on a single camera array, along with the surface of the bevel. Coating thickness of <1 μm was observed and ≈50 μm sized defects on the inner surfaces of the duct and on the bevel were identified. An IR transmission microscope set-up, which is automated to measure eight nozzles in ≈3 min, was constructed to measure coating coverage. The set-up offers a significant improvement in temporal resolution compared to the greater than ≈20 min needed for dye stain testing of a similar sized sample.

A test was developed to replace the standard industrial dye stain technique for identifying deficient coating. Inferior coating processes were used to produce consignments of samples with dye stain fail rates between 5% and 100%. These consignments were mixed with consignments containing zero dye stain fails. The IR transmission test was able to identify all consignments which produced failed samples and passed all the consignments of adequately coated samples. The two testing techniques had at times large discrepancies in the fail rate, with the IR test generally being higher. As dye stain testing is subjective, matching fail rates is difficult. A quantitative technique for measuring water dilution of the coating solution was also demonstrated and use of machine vision to identify machine malfunction was proposed.

Future work includes moving to the ≈1700 cm^−1^ region, where the PU cross-linker and PVP lubricant can be identified separately. Identification of separate absorption features also opens the prospect of measuring absolute coating thickness on the bevel. However, moving into the ≈1700 cm^−1^ region may require a cooled detector and/or a laser source, greatly increasing the cost of the set-up. Up-scaling the present set-up to efficiently measure coating coverage on increased quantities of nozzles, ≈10,000 per day, will be investigated and could enable movement away from random lot assurance sampling to entire product testing. Further adaption of the set-up can be made to measure coverage of other lubricants, such as PEG, PHM, and PSIT, as well as other bulk materials, such as PE and PC.

## Supplementary Materials

The following are available at https://www.mdpi.com/1424-8220/20/22/6408/s1.

## Abbreviations

The following abbreviations are used in this manuscript:

PE	poly(ethylene)
PC	poly(carbonate)
PP	poly(propylene)
PEG	polyethylene glycol
PHM	poly(hydroxyethyl methacrylate)
PSIT	poly(styrene-b-isobutylene-b-styrene)
PVP	poly(vinylpyrrolidone)
SEM	scanning electron microscopy
TEM	transmission electron microscopy
EDX	energy-dispersive X-ray spectroscopy
OCT	optical coherence tomography
ATR	attenuated total reflection
PU	polyurethane
PS	polystyrene
DOF	depth of field
U16	unsigned 16 bit
OD	optical density
SWIR	shortwave IR
MIR	mid IR

## References

[1] Maitz M. (2015). Applications of synthetic polymers in clinical medicine. Biosurf. Biotribol..

[2] Wypych G. (2016). Handbook of Polymers.

[3] Fotovvati B., Namdari N., Dehghanghadikolaei A. (2019). On Coating Techniques for Surface Protection: A Review. J. Manuf. Mater. Process..

[4] Bose S., Keller S., Alstrom T., Boisen A., Almdal K. (2013). Process optimization of ultrasonic spray coating of polymer films. Langmuir.

[5] (2017). Polypropylene Market Report Fourth Edition.

[6] Maddah H. (2016). Polypropylene as a promising plastic: A review. Am. J. Polym. Sci..

[7] Teodorescu M., Bercea M. (2015). Poly (vinylpyrrolidone)—A versatile polymer for biomedical and beyond medical applications. Polym. Plast. Technol. Eng..

[8] Akindoyo J., Beg M., Ghazali S., Islam M., Jeyaratnam N., Yuvaraj A. (2016). Polyurethane types, synthesis and applications—A review. RSC Adv..

[9] Murthy N., Damodaran V., Lee S., Hwang A., Sung H., Griesser H. (2016). Characterization of thin films for biomedical applications. Thin Film Coatings for Biomaterials and Biomedical Applications.

[10] Egerton R. (2005). Physical Principles of Electron Microscopy: An Introduction to TEM, SEM and AEM.

[11] Stout K., Blunt L. (2000). Three Dimensional Surface Topography.

[12] Smith E., Dent G. (2019). Modern Raman Spectroscopy: A Practical Approach.

[13] Mondal P., Diaspro A. (2013). Fundamentals of Fluorescence Microscopy: Exploring Life with Light.

[14] Smith B. (2011). Fundamentals of Fourier Transform Infrared Spectroscopy.

[15] Skoog D., Holler F., Crouch S. (2018). Principles of Instrumental Analysis.

[16] Alturkistani H., Tashkandi F., Mohammedsaleh Z. (2016). Histological stains: A literature review and case study. Glob. J. Health Sci..

[17] Kiernan J. (2006). Dyes and other colorants in microtechnique and biomedical research. Color. Technol..

[18] IARC Monographs Working Group (2010). General Introduction to the Chemistry of Dyes. IARC Monographs on the Evaluation of Carcinogenic Risks to Humans (VOLUME 99) Some Aromatic Amines, Organic Dyes, and Related Exposures.

[19] Walsh A., Barton K., Darby S., Wolfe R., Lewis L., McAuliffe M. (2019). Shortwave Infrared Imaging Of Thin Film Coatings Concealed Inside Polypropylene Tubing. Proceedings of the 10th Workshop on Hyperspectral Imaging and Signal Processing: Evolution in Remote Sensing (WHISPERS).

[20] Boyarchuk Y., Rappoport L., Nikitin V., Apukhtina N. (1965). A study of hydrogen bonding in urethane elastomers by infrared spectroscopy. Polym. Sci. USSR.

[21] Borodko Y., Habas S., Koebel M., Yang P., Frei H., Somorjai G. (2006). Probing the Interaction of Poly (vinylpyrrolidone) with Platinum Nanocrystals by UV-Raman and FTIR. J. Phys. Chem. B.

[22] Bradski G. (2000). The OpenCV Library. Dobb’s J. Softw. Tools.

[23] Azmi W., Sani R., Banerjee U. (1998). Biodegradation of triphenylmethane dyes. Enzym. Microb. Technol..

[24] Freiman D., Gall E. (1955). A staining method for the detection of polyvinylpyrrolidone (PVP) in tissue sections. Am. J. Clin. Pathol..

