# Supplementary material for: IR Imaging of Solid Lubricant Coatings on Concealed/Disjointed Surfaces for Transparent Polymer Delivery Device Applications
Source: Sensors (Basel). 2020 Nov 10;20(22):6408. doi: 10.3390/s20226408 (PMC7696912; doi:10.3390/s20226408)
Supplement: Supplementary file 1 [file sensors-20-06408-s001.zip › Supplementary material/Supplementary_Material.pdf]

IR imaging of solid lubricant coatings on concealed/disjointed surfaces for transparent polymerdelivery device applications

1. IMAGES OF CROPPING PROCEDURE

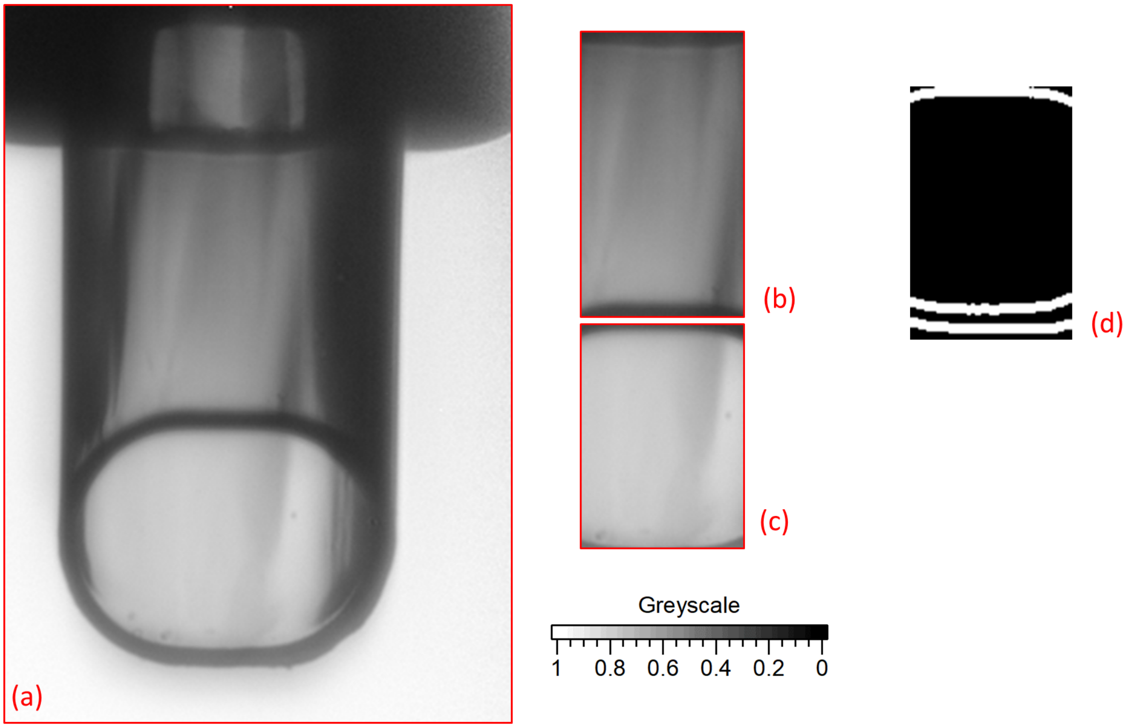

Figure S1. (a) Uncut U16 bit greyscale image of transmission at ≈ 3735 cm $^{-1}$. (b) Cropped to duct. (c) Cropped to bevel. (d) Contours determined on bevel using the *findContours* function of the OpenCV package, part of the python algorithm.

2. SEM IMAGES OF LOCAL FLUCTUATIONS IN COATING THICKNESS

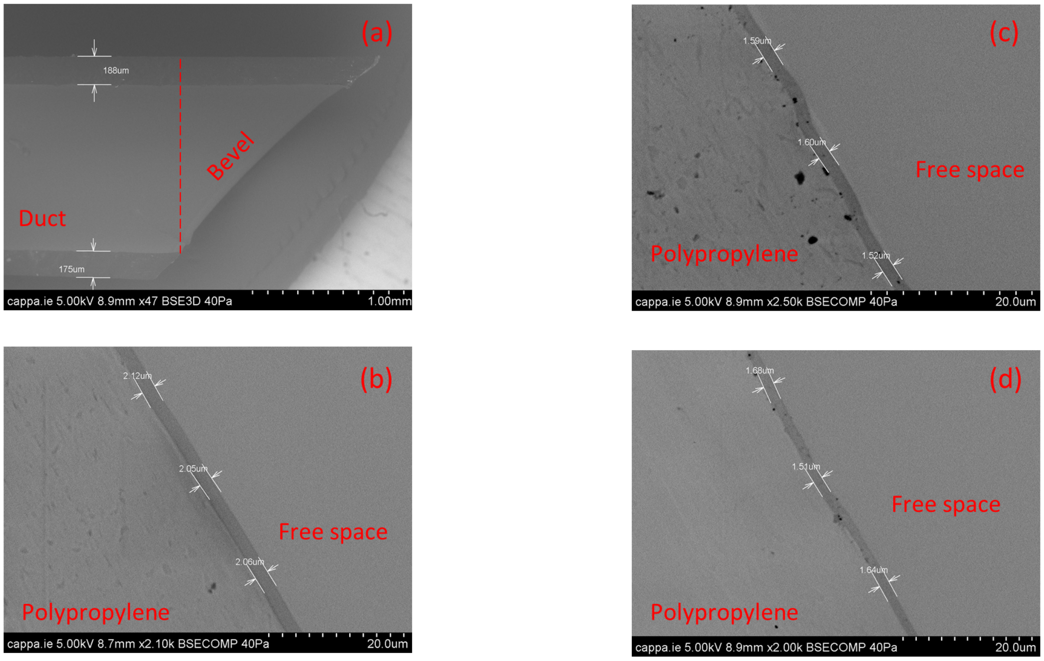

Figure S2. (a) Overview of nozzle cut. (b), (c) and (d) Sample coating thicknesses, over $\approx 50 - 60$ μm, beginning at drug entrance (b) and moving down duct towards bevel (c, d).
